# Retinal thinning in Gaucher disease patients and carriers: Results of a pilot study^☆^^☆☆^

**DOI:** 10.1016/j.ymgme.2013.04.001

**Published:** 2013-06

**Authors:** Alisdair McNeill, Gloria Roberti, Gerassimos Lascaratos, Derralynn Hughes, Atul Mehta, David F. Garway-Heath, Anthony H.V. Schapira

**Affiliations:** a Department of Clinical Neurosciences, UCL Institute of Neurology, UK; b NIHR Biomedical Research Centre at Moorfields Eye Hospital NHS Foundation Trust and UCL Institute of Ophthalmology, London, UK; c Lysosomal Storage Disorders Unit, Royal Free Hospital, London, UK

**Keywords:** Gaucher disease, Glucocerebrosidase, Optical coherence tomography, Parkinson's disease

## Abstract

Both Gaucher disease patients and heterozygous glucocerebrosidase mutation carriers are at increased risk of Parkinson's disease. Retinal thinning has been reported in early Parkinson's disease. Here we used optical coherence tomography to demonstrate thinning of the retinal ganglion cell layer in Gaucher disease patients and carriers who manifest clinical markers of potential early neurodegeneration. Optical coherence tomography may help identify Gaucher disease patients and carriers at increased risk of developing Parkinson's disease.

## Highlights

• Gaucher disease patients and carriers are at increased risk of Parkinson’s disease
• Retinal thinning is reported in Parkinson's disease
• Retinal thinning can be detected by optical coherence tomography (OCT)
• We utilized OCT to detect retinal thinning in Gaucher disease patients and carriers

## 1 Introduction

Mutations in the glucocerebrosidase gene (*GBA*), which encodes the lysosomal hydrolase deficient in Gaucher disease (GD), are the most common risk factor for Parkinson's disease (PD) [1]. Both GD patients and heterozygous *GBA* mutation carriers have a significantly increased risk of developing PD [1]. Clinical markers of potential early neurodegeneration include signs such as hyposmia, cognitive impairment and motor slowing, which have been associated with increased risk of developing a neurodegenerative disorder, chiefly PD [2]. However, it is acknowledged that these signs are neither sensitive nor specific for prodromal PD, with several non-neurological causes existing for hyposmia for example [2]. A subset of GD patients and carriers manifests clinical markers of potential early neurodegeneration, such as hyposmia and parkinsonian motor signs [3].

Detection of retinal thinning by optical coherence tomography (OCT) in PD has been described in multiple studies, which total several hundred patients and controls [4–9], with only a minority of studies reporting absence of retinal thinning in PD [10,11]. Retinal thinning correlates with disease severity in PD and may serve as a biomarker of disease progression in PD [4–9]. Retinal deposition of Gaucher cells has been described in GD, but there are no systematic studies of retinal pathology in this condition [12]. Here we utilized optical coherence tomography (OCT) to demonstrate retinal thinning in GD patients and heterozygous *GBA* mutation carriers who manifest clinical markers of potential early neurodegeneration.

## 2 Methods

Type I GD patients and heterozygous *GBA* mutation carriers, without a clinically manifest neurodegenerative disorder, and matched controls were recruited. Participants were assessed with the University of Pennsylvania Smell Identification Test (UPSIT), Unified Parkinson's Disease Rating Scale (motor subscale) and Montreal Cognitive Assessment (MoCA). OCT was performed using Fourier domain OCT (RTVue-100, Optovue Inc., Fremont, CA). OCT was performed with dilated pupils in both eyes, after ensuring that intra-ocular pressure was normal and visual fields were full. The ganglion cell complex thickness (GCC), which extends from the internal limiting membrane to the inner nuclear layer, was measured as a marker of retinal thinning. Mean GCC thickness was compared between groups using a one way ANOVA test and post hoc testing with least squares difference test (IBM, PASW version 20.1). The most severely affected eye from each individual was analysed. The most severely affected eye was defined as that with the thinnest GCC measurement. GD patients and carriers were split into 2 groups: 1 group who had potential clinical markers of prodromal neurodegeneration and 1 group without abnormalities of these clinical markers. The non-neurological controls were a third group.

### 2.1 Patients

Eleven GD patients (5 female, mean age 63.7 years, range 45–89 years, 6/11 N370S/N370S genotype and remainder N370S/L444P), 3 *GBA* mutation carriers (2 female, mean age 70.6 years, all N370S genotype) and 7 controls with no neurological disease matched for age (mean age 67, range 51–85, p = 0.9 vs GD) and sex (4 female) were studied. No study participant had evidence of retinopathy or glaucoma on ophthalmoscopy and all had normal intraocular pressures to exclude glaucoma. The GD patients had clinical features typical of Type I disease such as visceromegaly, bone disease and blood dyscrasia but no eye movement disorders. Four GD patients had clinical markers of potential early neurodegeneration: 1 with hyposmia (UPSIT score under 15th centile for age and sex), 1 with cognitive impairment (MoCA score 23), 1 with hyposmia and cognitive impairment, 1 with parkinsonian motor signs (rest tremor and rigidity but no bradykinesia, which therefore does not meet Queen Square Brain Bank criteria for a diagnosis of Parkinson's disease). Two carriers had hyposmia. None of the study participants were smokers or had known disease of their upper airways or head injury which might cause olfactory impairment. The age of the GD patients and carriers with and without clinical markers of potential early neurodegeneration did not differ (62.5 +/− 3.7 years versus 65.0 +/− 14 years, p = 0.81).

### 2.2 Optical coherence tomography results

**Fig. 1:**
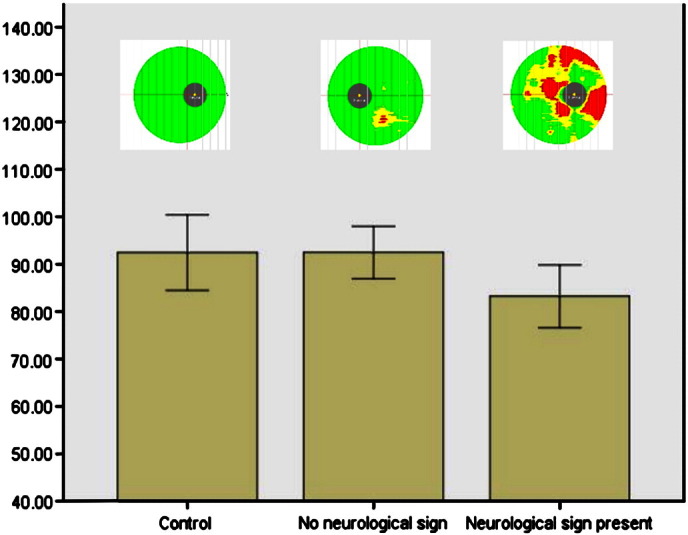
Mean ganglion complex thickness as measured by optical coherence tomography. The mean ganglion complex thickness was significantly lower in the group of Gaucher disease patients and GBA mutation carriers who had clinical markers of potential early neurodegeneration (bar marked neurological sign present) than in the control group or the group of Gaucher disease patients and carrier without clinical markers of potential early neurodegeneration (bar marked no neurological sign). Above each bar is a representative heat map of the optical coherence tomography studies. Red areas represent ganglion complex thickness less than 1% of predicted for age and sex, yellow areas less than 5% of predicted and green areas thickness greater than 5% of predicted. The grey disc represents the fovea.

One way ANOVA demonstrated a significant difference in GCC thickness between groups (F = 4.01, p = 0.036). The mean GCC thickness for GD patients and carriers without potential clinical markers of early neurodegeneration (mean 92.4 +/− 5 μM) did not differ from controls (mean 93.4 +/− 8 μM, p = 0.9). Mean GCC thickness for GD patients and heterozygous *GBA* mutation carriers with potential clinical markers of early neurodegeneration (83.2 +/− 6 μM) was significantly less than that of controls (p = 0.025) or GD patients and carriers without potential clinical markers of early neurodegeneration (p = 0.021) (Fig. 1). The difference in the mean GCC thickness remained significant after correcting for the participants' refractive error.

## 3 Discussion

Here we provide in vivo OCT data showing retinal thinning in GD and heterozygous *GBA* mutation carriers. The mechanisms leading to retinal degeneration in GD and carriers are unclear. Work in our lab has demonstrated that loss of glucocerebrosidase enzyme activity is associated with oxidative stress and mitochondrial dysfunction [13], which can be associated with retinal degeneration. Autophagy has been reported to play a role in retinal ganglion cell death in a murine model of the lysosomal storage disorder Niemann–Pick disease type C [14]. Thus, a role for lysosomal pathology in retinal degeneration warrants further investigation. Several studies have documented thinning of the retina, including ganglion cell loss, in PD [4–9]. In our cohort retinal thinning is associated with clinical markers of potential early neurodegeneration in GD patients and heterozygous *GBA* mutation carriers. This suggests that retinal thinning detected by OCT may serve as a biomarker of increased risk of developing a neurodegenerative disorder, such as PD, in this cohort. However, this needs to be proven in larger, prospective trials. Clinicians should be aware that GD and carriage of heterozygous *GBA* mutations can be associated with retinal thinning and should include these in the differential diagnosis of retinal pathology when appropriate.

## Author roles, funding and conflicts of interest

Alisdair McNeill—Study design, study performance, drafting and editing of manuscript, statistical analysis. Funded by a United Kingdom Medical Research Council Training Fellowship and no conflicts of interest.

Gloria Roberti—Data collection (performed optical coherence tomography), no conflicts of interest.

Gerassimos Lascaratos—Data collection, editing of manuscript. Funded by the NIHR Biomedical Research Centre at Moorfields Eye Hospital NHS Foundation Trust and UCL Institute of Ophthalmology, Fight for Sight and Allergan Europe. No conflicts of interest.

Derralyn Hughes—patient recruitment, editing manuscript. No conflicts of interest.

Atul Mehta—patient recruitment, editing manuscript. No conflicts of interest.

David Garway-Heath—Study design, revising manuscript. Receives funding from the National Institute for Health Research (UK) Biomedical Research Centre at Moorfields Eye Hospital and the UCL Institute of Ophthalmology. Professor Garway-Heath's chair at UCL is supported by funding from the International Glaucoma Association. No conflicts of interest.

Anthony Schapira—Study design, revising manuscript. This work was supported in part by the Wellcome Trust/MRC Joint Call in Neurodegeneration award (WT089698), UK Parkinson's Disease and Kattan Trust. No Conflicts of interest.
