# Corrigendum to “Retinal thinning in Gaucher disease patients and carriers: Results of a pilot study” [Mol. Genet. Metab. 109 (2013) 221–223]

**DOI:** 10.1016/j.ymgme.2013.12.297

**Published:** 2014-03

**Authors:** Alisdair McNeill, Gloria Roberti, Gerassimos Lascaratos, Derralynn Hughes, Atul Mehta, David F. Garway-Heath, Anthony H.V. Schapira

**Affiliations:** a Department of Clinical Neurosciences, UCL Institute of Neurology, UK; b NIHR Biomedical Research Centre at Moorfields Eye Hospital NHS Foundation Trust and UCL Institute of Ophthalmology, London, UK; c Lysosomal Storage Disorders Unit, Royal Free Hospital, London, UK; d IRCCS-Fondazione GB Bietti, Rome, Italy

At the time of the study I was working in London at Moorfields Eye Hospital as a research fellow, sponsored by IRCCS-Fondazione GB Bietti, Rome, Italy where I was employed.

These are the details: IRCCS-Fondazione GB Bietti, Via Livenza 3, 00198 Rome, Italy.

